# Clonal Analysis of Gliogenesis in the Cerebral Cortex Reveals Stochastic Expansion of Glia and Cell Autonomous Responses to Egfr Dosage

**DOI:** 10.3390/cells9122662

**Published:** 2020-12-11

**Authors:** Xuying Zhang, Christine V. Mennicke, Guanxi Xiao, Robert Beattie, Mansoor A. Haider, Simon Hippenmeyer, H. Troy Ghashghaei

**Affiliations:** 1 Department of Molecular Biomedical Sciences, College of Veterinary Medicine, North Carolina State University, Raleigh, NC 27607, USA; xzhang39@ncsu.edu (X.Z.); skaysiu@gmail.com (G.X.); 2 Department of Mathematics, North Carolina State University, Raleigh, NC 27695, USA; cvmennic@ncsu.edu (C.V.M.); m_haider@ncsu.edu (M.A.H.); 3 Institute of Science and Technology Austria, Am Campus 1, 3400 Klosterneuburg, Austria; robert.beattie@ist.ac.at (R.B.); Simon.Hippenmeyer@ist.ac.at (S.H.)

**Keywords:** cerebral cortex, clonal analysis, neurogenesis, gliogenesis, Egfr, astrocyte, oligodendrocyte MADM, stochastic, deterministic

## Abstract

Development of the nervous system undergoes important transitions, including one from neurogenesis to gliogenesis which occurs late during embryonic gestation. Here we report on clonal analysis of gliogenesis in mice using Mosaic Analysis with Double Markers (MADM) with quantitative and computational methods. Results reveal that developmental gliogenesis in the cerebral cortex occurs in a fraction of earlier neurogenic clones, accelerating around E16.5, and giving rise to both astrocytes and oligodendrocytes. Moreover, MADM-based genetic deletion of the epidermal growth factor receptor (Egfr) in gliogenic clones revealed that Egfr is cell autonomously required for gliogenesis in the mouse dorsolateral cortices. A broad range in the proliferation capacity, symmetry of clones, and competitive advantage of MADM cells was evident in clones that contained one cellular lineage with double dosage of Egfr relative to their environment, while their sibling *Egfr-null* cells failed to generate glia. Remarkably, the total numbers of glia in MADM clones balance out regardless of significant alterations in clonal symmetries. The variability in glial clones shows stochastic patterns that we define mathematically, which are different from the deterministic patterns in neuronal clones. This study sets a foundation for studying the biological significance of stochastic and deterministic clonal principles underlying tissue development, and identifying mechanisms that differentiate between neurogenesis and gliogenesis.

## 1. Introduction

Mechanisms that regulate the equilibrium between stem cell expansion and differentiation are critical to balanced production of neurons and glia for homeostatic brain function. Neurogenesis in the cerebral cortex may yield finite numbers of neurons governed by a deterministic clonal rule [1]. Accordingly, cortical progenitor cells generate prescribed and predictable numbers of neurons in a precise window during embryonic development. This differs from regenerating tissues that maintain progenitors throughout adulthood (e.g., skin, liver, lung epithelium and the gastrointestinal tract), where the balance between expansion and differentiation at the level of individual clones appears stochastic [2,3,4,5]. Curiously, dynamics of expansion and differentiation at the level of total cellular populations within these tissues balance out in predictable patterns (i.e., total number of cells produced, maintenance of lineage patterns, and ultimately the shape of the tissue they form). In this context, the cortex loses its ability to regenerate after embryogenesis, suggesting that clonal rules may be different in organs that fail to regenerate during adulthood versus continuously regenerating tissues. 

Gliogenesis begins during late neurogenesis, is a critical aspect of brain homeostasis, and unlike neurogenesis it remains active and responsive to injury and disease for life [6,7,8,9,10]. Mature glial cells comprise major subtypes based on their function, morphology and embryonic origins. Macroglia are distinct from microglia in their ontogeny; macroglia are generated from CNS progenitors (neuroectodermal origin), whereas microglia are derived from the hematopoietic system (mesodermal derived) around midgestation [11]. Macroglia consist of astrocytes and oligodendrocytes, which are distributed throughout the white and grey matters of the mature brain, are regionally and molecularly diverse [12,13,14], and act as supporting cells for various neural networks. Only a few studies have addressed the clonal aspects of gliogenesis, some focusing largely on oligodendrocyte production [15], while others have focused on astrocytogenesis [16,17,18,19,20]. These studies reported on widespread clones, which the investigators interpreted as heterogeneous in their origin and possibly function. However, the imprecise and invasive nature of the broadly used embryonic electroporation or viral infection approaches in the past studies, and the high density of labeled cells in the cortex with these methods, leave their interpretive power regarding clonality of cells limited. 

Here we report on a detailed analysis of gliogenesis during various stages of embryonic development at a single postnatal time point in mice. To test the effects of disrupting gliogenesis on overall neuronal and glial clones, we focused on manipulating the expression of the epidermal growth factor receptor (Egfr). While developmental function of Egfr has been studied in various contexts since the first report on its germline deletion [21] (these mice are perinatal lethal), its precise role in the development of the CNS remains unclear. Gliogenesis is certainly under the influence of Egfr and its predominant ligand Egf during perinatal development [22,23,24,25]. While a number of studies have implicated Egfr signaling in oligodendrocyte development within the forebrain [24,25,26,27,28,29], we and others have suggested a role in astrocyte production [30,31,32,33], and in neurogenesis and neuronal survival [23,34,35]. Thus, Egfr’s role in gliogenesis and neurogenesis remains unclear and no information is available on its functions at the clonal level.

## 2. Materials and Methods

### 2.1. Animals

Mice were used under the regulations and approval from the Institutional Animal Care and Use Committee at North Carolina State University. Mice were housed in a 12-h light:dark cycle with ad libitum access to food and water. Mice for mosaic analyses were generated using breeding schemes that have been previously described [36]. Briefly, *Egfr floxed* mice [37] were bred to Mosaic Analysis with Double Markers (MADM)-11^TG^ mice (*Igs2^tm2(ACTB-TdTomato,-EGFP)Luo^*/J, The Jackson Laboratory, Bar Harbor, USA; #013751) to generate *Egfr^F/F^*; *MADM-11^TG/TG^* mice via meiotic recombination. *MADM-11^GT/GT^* mice (*Igs2^tm1(ACTB-EGFP,-tdTomato)Luo^*/J, The Jackson Laboratory, #013749) were bred to *Nestin-creERT2* mice (Tg(Nes-cre/ERT2)1Fsh) to generate *Nestin-creER; MADM-11^GT/GT^* mice. For experiments, *Egfr^F/F^; MADM-11^TG/TG^* mice were crossed to *MADM-11^GT/GT^* mice with the *Nestin-creERT2* allele. Control mice were generated by crossing *Nestin-creERT2; MADM-11^GT/GT^ mice to MADM-11^TG/TG^* mice. *Egfr floxed* mice were genotyped for the presence of the wild type (*WT*) and *floxed* alleles using the primers: Egfr lox3 F: 5′ CTTTGGAGAACCTGCAGATC; Egfr lox3 R: 5′ CTGCTACTGGCTCAAGTTTC. All other mouse lines (MADM alleles and *Nestin-creERT2*) were genotyped using protocols provided by Jackson Laboratories. For induction of cre-mediated recombination, Tamoxifen (TAM; Millipore Sigma, St. Louis, USA, 10 mg/mL in corn oil) was administered to timed pregnant dams at 50 micrograms/gram body weight by oral gavage via plastic feeding tube (Instech, Plymouth Meeting, USA). 

### 2.2. Tissue Processing and Immunohistochemistry

Pregnant dams were sacrificed by Avertin overdose (2,2,2 tribromoethanol; 7.5 mg/g body weight) followed by cervical dislocation around E20 just prior to birth and embryos were extracted and transferred to a surrogate female. Pups were allowed to survive to postnatal day 30 (P30) when they were perfused with 4% paraformaldehyde (PFA) in phosphate buffer saline (PBS, 0.1 M, hereafter), and brains were dissected and submerged in 4% PFA in PBS at 4 °C overnight. Brains were embedded in 3% low melting point DNA-grade agarose in PBS and serial 50 μm sections were collected using a vibratome (Leica VT1000S, Leica, Buffalo Grove, USA).

Floating serial sections were washed with PBS and blocked for 1 h at room temperature in blocking buffer (10% normal donkey or goat serum, 1% Triton X-100, PBS). Sections were incubated with primary anti-GFP (Abcam, Cambridge, MA; ab13970, 1:2000) and Rabbit anti-RFP (Abcam, ab62341, 1:500) antibodies diluted in 0.1% blocking buffer overnight at 4 °C, followed by 3 5-min washes with PBS at room temperature the next day. AlexaFluor goat anti rabbit Cy3 (Thermo Fisher Scientific, Waltham, USA; A10520, 1:1000), AlexaFluor goat anti-chicken 488 (Thermo Fisher Scientific, A11039, 1:1000) secondary antibodies were diluted in blocking buffer and incubated with the serial sections for 1 h at room temperature, followed by 3 washes with PBS. Sections were counterstained with the DNA marker (4′,6-diamidino-2-phenylindole; DAPI) at 1:2000 during the secondary incubation. Sections were mounted onto glass slides and coverslipped with Faramount aqueous mounting medium (Dako, Agilent Technologies, Santa Clara, USA). Images were acquired using an FV1000 confocal microscope (Olympus, Waltham, USA). 

### 2.3. Quantifications and Statistical Analyses

Captured clones were analyzed by counting the number of red and green cell types in the cortical parenchyma in serial confocal images. Counts were restricted to dorsolateral motor, somatosensory, and parietal cortices (including areas MOs, MOp, SSp, SSs, PTLp annotated in the Allen Brain Atlas, https://mouse.brain-map.org/experiment/thumbnails/100048576?image_type=atlas). Counts were recorded in Excel and standard statistical methods were employed for general analyses. Categorization of clones by type and symmetry and subsequent statistical tests between categories of clones were performed in MATLAB (version R2018a; MathWorks, Natick, USA). Clone types were assigned using logical indexing to identify which clones had zero glia (N), zero neurons (G), or positive numbers of both cell types (Mix). For symmetry categories in neural-containing clones, logical indexing was used to identify which clones had >3 neurons in both red/green lineages (symmetric), 1–3 neurons in one lineage and >3 neurons in the other lineage (two-sided asymmetric), or 1–3 neurons in both lineages (small). A similar process was performed for glia in glia-containing clones. One-sided asymmetric clones were identified with logical indexing to find whether the sum of red cells or sum of green cells in a clone was equal to zero. A portion of Mix clones did not fall into any of these categories—these clones had neurons in only one MADM lineage and glia in the other MADM lineage (or in both colors). These clones fail to be categorized as one-sided since they have cells in both red and green lineages, despite having zero neurons or zero glia on one side. We opted to groups these clones into the two-sided asymmetric category since their production of neurons or glia is asymmetric but their overall composition is not restricted to one color.

All data were presented as numbers of cells per clone, or as mean and standard error of the mean (sem). Significance was evaluated by Student’s t-tests, univariate ANOVA, Wilcoxon Rank-sum, and Chi square tests with *p*-values of less than or equal to 0.05 considered significant.

### 2.4. Computational Models

Two types of models from previous studies were used as a basis for identifying deterministic or stochastic patterns in the distributions of neurons or glia per clone. One study found that asymmetrically differentiating progenitors produced a normally distributed number of neurons (averaging ~8 neurons per clone), yielding a population-level multi-Gaussian distribution with peaks at integer multiples of the average [1]. The starting point for our model is the corresponding clone size distribution:

(1) $$M_{CON}\left(i|\vec{p}\right)=\sum_{j=1}^{k}\beta_{j}^{2}N\left(i|j\mu_{0},\sigma_{j}^{2}\right),$$

which, due to the nature of the clonal expansion, was described as “deterministic” [1]. Here, *k* is the number of Gaussians in the model, $\vec{p\;}$ is the vector of parameters $\left[\beta_{j}^{2},\mu_{0},\sigma_{j}^{2}\right]$, $\beta_{j}^{2}<1$ is a positive weight, and $N(i|j\mu_{0},\sigma_{j}^{2})$ is a normal distribution with mean $j\mu_{0}$ and variance $\sigma_{j}^{2}$ evaluated at integers *i*. 

Neural production can also be modeled using a Galton–Watson stochastic branching process [38] in which the two offspring of a dividing progenitor are randomly determined according to probabilities p (of producing two neurons) and q (of producing two progenitors); thus 1−p−q is the probability of producing one neuron and one progenitor. This type of stochastic process produces a distribution of clone sizes where the probability of a clone of size n is

(2) $$Q_{2}=p,\;Q_{3}=p\left(1-p-q\right),$$

(3) $$Q_{n}=q\sum_{k=2}^{n-2}Q_{k}Q_{n-k}+\left(1-p-q\right)Q_{n-1},\;\;\;n\geq 4$$

which resembles a geometric distribution whose shape is dependent on *p* and *q* [38].

In addition to these existing models, we formulated two new models for quantitative analysis of glial clone sizes (https://repository.lib.ncsu.edu/handle/1840.20/38042). First, expanding upon the prior stochastic model [38], we formulated a stochastic branching process model with two stages of clonal expansion. This extension represents a stochastic clonal mechanism involving a progression from progenitors to intermediate progenitors (IP) to glia. Here, a progenitor produces another progenitor and an IP offspring according to probabilities $p_{1}$ and $q_{1}$, where the former is the probability of producing two IPs and the latter is the probability of producing two progenitors; thus $1-p_{1}-q_{1}$ is the probability of producing one IP and one progenitor. IP cells then divide to produce IPs and glia in a similar manner; here $p_{2}$ is the probability of producing two glia, $q_{2}$ is the probability of producing two IPs, and $1-p_{2}-q_{2}$ is the probability of producing one IP and one glial cell. We derived a similar formula for the probability of a clone of size *n* arising from this two-stage process:

(4) $$Q_{n}=\sum_{m=2}^{16}Q_{n}^{m},\;\;\;n\geq 4$$

where

(5) $$Q_{n}^{m}=\left(1-p_{1}-q_{1}\right)\sum_{k=2}^{n-2}Q_{k}^{m-1}Q_{n-k}^{1}+q_{1}\sum_{k=2}^{n-2}\sum_{l=2}^{m-2}Q_{k}^{l}Q_{n-k}^{m-l},$$

where m is the number of IP cells produced from the initial progenitor. A maximum value of m=16 was used to model the switch from progenitors to IPs in relatively few generations. If m>16, at least four generations of progenitor divisions are required to produce m IP cells.

(6) $$M_{UNCON}\left(i|\vec{p}\right)=\sum_{j=1}^{k}\beta_{j}^{2}N\left(i|\mu_{j},\sigma_{j}^{2}\right).$$

The second new model extends $M_{CON}$ above, relaxing the requirement of equally spaced peaks,

This model was formulated primarily for hypothesis testing purposes, noting that $M_{CON}$ is nested within $M_{UNCON}$, and thus the two models can be compared to one another using an F-test [39]. Specifically, we were able to test whether relaxing the constraint of equally spaced peaks produces a better fit to the distribution of clone sizes, thus supporting or negating attribution of the deterministic mechanism proposed in Gao et al. [1] to glia. Alternative mechanisms consistent with unequally spaced peaks were not immediately identifiable, but a couple of possibilities exist. For instance, glial production could be Gaussian but multi-modal, pointing to multiple deterministic patterns. Alternatively, a combination of stochastic and deterministic behaviors could be present, which would obscure the regularity of the Gaussian peaks.

## 3. Results

### 3.1. Astrocytic Clones Are Responsive to Dosage of Egfr in Dorsolateral Cortices at P30

**Figure 1:**
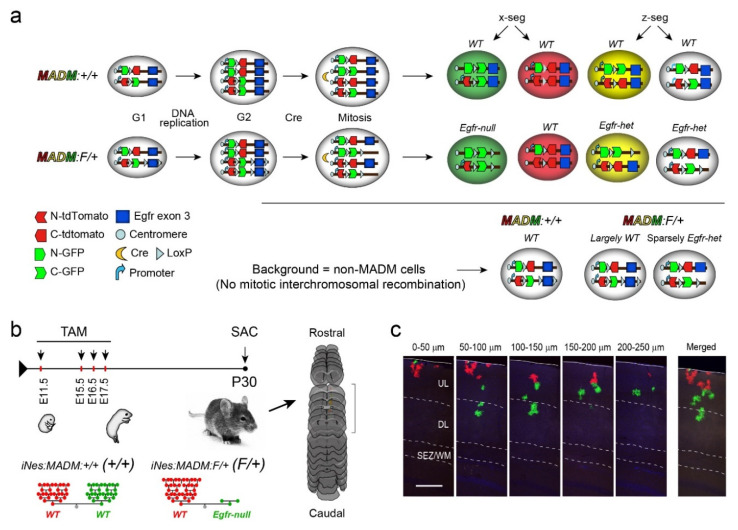
Clonal Analysis of Gliogenesis using Mosaic Analysis with Double Markers (MADM). (**a**) Molecular basis of labeling individual progenitor clones with distinct fluorescent reporters (GFP, green and tdTomato, red). Cre mediated interchromosomal mitotic recombination is followed by two distinct segregation patterns (x-seg and z-seg) generating clonal siblings in distinct colors as indicated. In control MADM mice, all cells will be *WT* and referred to as +/+. When a single floxed allele for Egfr is introduced, green cells lack Egfr (*Egfr-null*), while the red cells are maintained as *WT* (F/+ mice). Background genotypes obtained from the described genetic combinations using a tamoxifen induced *Nestin-creERT2* transgene result in substantial differences in the *Egfr* genotype in background cells (non-MADM recombination events) which exert non-autonomous effects on MADM cells. (**b**) Schematic of tamoxifen (TAM) induction time points during embryonic development, and processing of forebrains in serial sections. Inductions were performed in *iNes:MADM:Egfr ^+/+^* (*+/+*) and *iNes:MADM:Egfr ^F/+^* (*F/+*) mice, which result in the generation of clones containing green and red cells that become distinguished following TAM inductions. Green MADM F/+ glia (*Egfr-null*) fail to develop, while glial production is elevated in the red sibling’s population. (**c**) Confocal images of a rare symmetric “G” cortical clone in an E16.5 induced +/+ cortex. SEZ/WM, subependymal zone/white matter; DL, deep layers; UL, upper layers. Scale bar, 100 µm.

To conduct clonal analysis of gliogenesis in vivo without the use of invasive approaches, we used sparse labeling of progenitors at clonal densities in mice with Mosaic Analysis with Double Markers [36] enabled on the mouse chromosome 11 (MADM-11) [40,41]. MADM operates through two reciprocal chimeric reporter genes inserted near centromeric regions of chromosomes (here mouse chromosome 11; Figure 1a). Cre-mediated interchromosomal recombination events result in definitive labeling of cells at low densities with two segregation patterns. EGFP (green) and tdTomato (red) expressing cells originate from one type of division whereas yellow and non-fluorescent siblings are derived from another type of division (Figure 1a). 

To achieve Egfr deletion in MADM neural progenitor clones, we used two sets of MADM mice; one where all MADM labeled cells are *wild type* (*WT*; *iNes:MADM:Egfr ^+/+^* mice referred to as *+/+* herein), and another in which a *floxed Egfr* allele is crossed into the MADM background resulting in siblings with differential dosages of Egfr by carrying *WT* (tdTomato^+^, red), heterozygous (*Egfr- het*; tdTomato^+^/EGFP^+^, yellow), and homozygous (*Egfr-null*; EGFP^+^, green) floxed alleles of Egfr at low densities (*iNes:MADM:Egfr ^F/+^*, referred to as *F/+* herein, Figure 1a). A *Nestin-creERT2* transgenic mouse [42] was used to induce the labeling of MADM clones in a temporally defined manner during the gliogenic period using tamoxifen (TAM) injections in time-pregnant dams. Pups were allowed to survive to postnatal day 30 when the analysis of clones was conducted in serial brain sections (P30; Figure 1b). Because the MADM recombination event results in permanent labeling of the daughter cell and its progeny from subsequent cell divisions, we were able to unambiguously track genetically manipulated cells and fate specification in clonally derived daughter cells at postnatal day 30 (P30; Figure 1b). Due to the presence of a single *Egfr floxed* allele in all cells of F/+ mice, we assume the presence of a population of unlabeled *Egfr-het* cells that are intermingled with the MADM-labeled cells (Figure 1a). MADM-11 animals without the floxed allele for Egfr were used as controls (+/+ mice; all three combinations of reporter^+^ cells are *WT*). 

**Figure 2:**
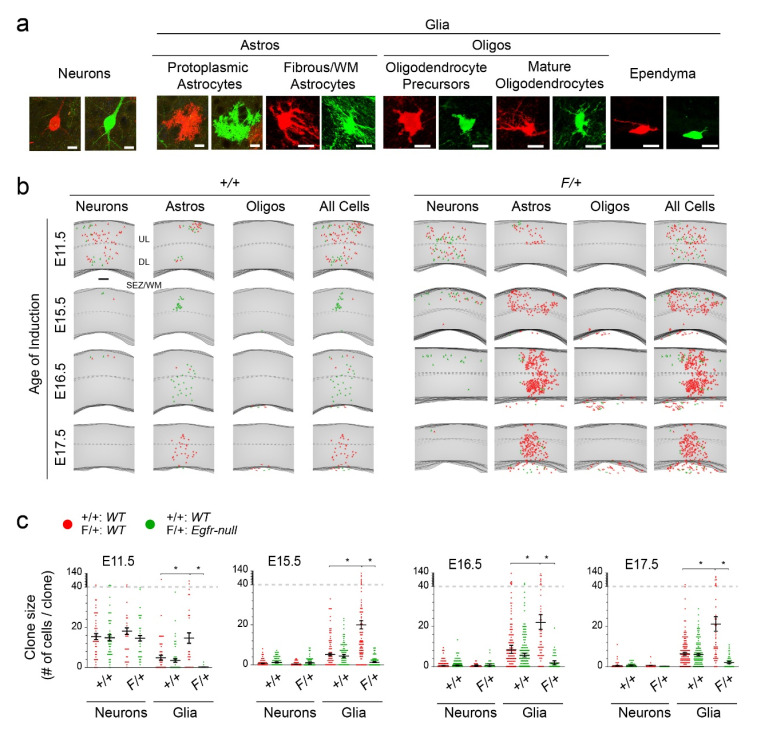
Glial subtypes in MADM clones. (**a**) Samples representing classified cell types in both red and green MADM cells. Scale bars, 10 µm. (**b**) Maps of cell type distributions in the cortex of +/+ and F/+ mice at different induction time points. Scale bars, 100 µm. (**c**) Plots of clone sizes (number of total cells) in the green and red clonal populations in the cortex at P30 under various induction time points. Each dot represents individual clones and averages are depicted ± sem. *, Student’s t-test, *p* < 0.05.

We focused on clones containing green (Gfp+) and red (tdTomato+) MADM cells as they inform on symmetry of clonal expansion under *WT* conditions in +/+ mice, as well as containing an Egfr dosage model in F/+ cortices. Cells were counted in a total of 439 +/+ clones and 203 F/+ clones and phenotyped as either neurons or glia based on morphological characteristics (Figure 2a). Counting was restricted to clones in the dorsolateral cortices to avoid regional, developmental, and structural variabilities across cortical areas. Analyses were confined to clonal cell counts in the upper and deep layers (layers 1–6) of the cortex and do not include data from the underlying white matter (WM) and the subependymal zone (SEZ), which contain complex sets of cells including active neural and glial progenitors at P30. In a fraction of clones where we conducted a detailed survey of cell types including the SEZ/WM (40 clones per induction time, per genotype), the majority of cells in upper and deep layers of the cortex were astrocytes (Figure S1). Only a few oligodendrocyte-containing clones in the +/+ brains were found in the deepest parts of layer 6 (percentages of all clones at each induction age: 10.9% at E11.5, 0% at E15.5, 0% at E16.5, 2.2% at E17.5; Figure S1). These fractions were elevated in the SEZ/WM where robust numbers of oligodendrocyte precursors reside (17.4% at E11.5, 32.6% at E15.5, 39.1% at E16.5, 28.3% at E17.5; Figure S1). A very small portion of clones also contained ependymal cells which often appeared as pairs of red and green MADM cells (Figure S1), suggesting their likely derivation from a terminal differentiative symmetric division. The fractions of oligodendrocytes in the F/+ cortices were mostly similar to those in the +/+ cortices across the different induction times (Figure S1). Thus, the majority of cells labeled as “glia” in this study are astrocytes in the cortical layers II–VI. Details on the SEZ/WM cells, and additional characterization of subclasses of astrocytes in the cortical layers, requires further classifications with the aid of molecular markers (subject of a separate study). Nevertheless, within the cortical plate (layers 1–6) several combinations were observed (Figure S2). At E11.5, purely neuronal clones were present while they became less prevalent at E15.5–E17.5. Moreover, neuron plus oligodendrocyte containing clones were only present in E11.5 clones, but not the others. Purely astrocytic clones emerge at E15.5–E17.5, while we failed to observe any clones only containing oligodendrocytes within the cortical plate at any time point. F/+ cortices contained similar distributions of cells (when red and green cells are combined), although their proportions were changed. For example, astrocyte-containing clones became far more abundant in F/+ cortices compared to +/+ clones (Figure S2). 

Plotting of clone sizes and cell types across time points matched the standing paradigm that gliogenesis accelerates during E15.5–E17.5 with larger clones containing glia compared to earlier TAM inductions during neurogenesis (Figure 2b,c). Moreover, in the F/+ cortices there was a significant increase in number and percentages of red *WT* glia concomitant with the near complete abolition of green *Egfr-null* glia, both of which reside in an *Egfr-het* background (Figure 2b,c). In this dosage model, it became clear that Egfr is sufficient (via analysis of red MADM cells in *F/+* cortices) and required (via analysis of green MADM cells in *F/+* cortices) for induction and/or expansion of a glial fate in the dorsolateral cortices. 

Laminar positioning of clonal neurons in the upper (UL) and deep (DL) layers of the +/+ dorsolateral cortices also followed the established developmental patterns of cortical neuronal layering (DL early, UL late; Figure S3). Accordingly, neuron production was proportionally similar in UL and DL in E11.5-induced +/+ cortices, but more UL neurons were quantified in E15.5, E16.5, and E17.5 clones. In comparison, astrocyte production was similar in UL and DL in both E11.5 and E15.5 clones, with more astrocytes found in the DL at E16.5, and more UL astrocytes at E17.5 in +/+ cortices. As described earlier, oligodendrocytes were predominately found in the DL, with almost none found in the UL. In comparison, F/+ clones exhibited significant disruptions in the laminar positioning of both neurons and glia in F/+ clones, with more UL neurons found in E11.5, E15.5, and E16.5 inductions, whereas few (if any) neurons could be found in E17.5 clones. In F/+ clones, the average number of red (*WT*) UL neurons was significantly greater and more variable than the average number of green (*Egfr-null*) UL neurons at E11.5, whereas the opposite pattern was consistently produced at the later induction times (greater numbers of green UL neurons than red UL neurons). This finding suggests that the double dosage of Egfr in red WT F/+ MADM cells, relative to their environment, facilitates the final rounds of neuronal production resulting in their depletion, such that almost no UL red neurons are observable in F/+ clones inducted after E15.5 (also see later). Interestingly, total astrocyte production in F/+ clones failed to exhibit the same pattern seen in +/+ clones with no differential distributions found between UL and DL at any time (Figure S3). Oligodendrocytes were restricted to the DL in F/+ clones, as they were in +/+ cortices; however, red MADM oligodendrocytes were significantly more abundant than green cells in F/+ sublineages (Figure S3). Thus, layering of astrocytes appears to largely follow the inside-out pattern seeding of neurons with a delay, and the F/+ genotype disrupts this pattern with no discernable differences in UL and DL positioning.

### 3.2. Distinct Clone Types and Their Responsiveness to Egfr Dosage in the Dorsolateral Cortices

**Figure 3:**
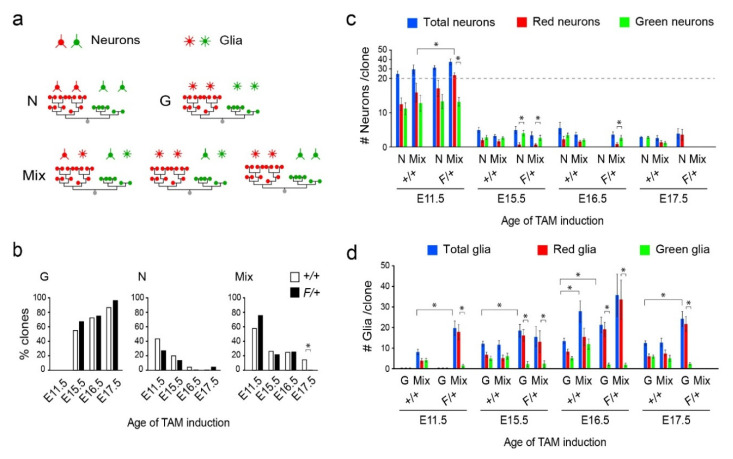
Analysis of MADM clone types. (**a**) Classification of clones based on their neuronal and glial compositions. (**b**) Percentage of all clones containing G, N or Mix types across the different induction time points and genotypes (green and red MADM cells are combined). (**c**) Numbers of neurons in different clone types in +/+ and F/+ cortices at different induction times. Total numbers of neurons are broken down into their green and red sibling fractions as shown. The scales for *Y*-axis values are not the same for the 0–20 and 20–50 ranges in order to reveal the low values for neurons in E15.5–E17.5 induced clones. (**d**) Numbers of glia in different clone types in +/+ and F/+ cortices at different induction times. Total numbers of glia per clone are broken down into their green and red sibling fractions as shown. Data are mean ± sem. *, rank-sum test, *p* < 0.05.

Beyond general observations based on standard statistical analyses, MADM uniquely provides data on clone sizes (total cells per clone) and clone symmetries (relative cell counts in red and green lineages), in addition to cell type information. As such, the multidimensional MADM datasets require in-depth computational analyses of the relationship between total clonal output and the fates of the two daughter lineages. Clones were first categorized as containing only neurons (N), only glia (G), or a mix of the two (Mix), regardless of whether the MADM cells were red or green (Figure 3a). Results revealed a clear distinction in the percentages of clone types across the different induction time points (Figure 3b). Aligned with emergence of gliogenesis late during cortical development, a significant number of G clones were found in the cortex of E15.5, E16.5, and E17.5 TAM-induced embryos, whereas none could be found in E11.5 clones. This finding suggests the absence of glia-restricted progenitors in the early developing cortex and that glia largely originate from previously neurogenic lineages. Overall, these findings are consistent with the lineage-progression model of cortical development [1,43,44,45], and match our previous report on the deleterious effects of *Egfr* deletion on gliogenesis in MADM populations in the cortex [33]. Mix clones were present more often in F/+ than +/+ cortices at E11.5 (but not at E15.5 or E16.5; Figure 3b). However, there were significantly fewer F/+ Mix clones than +/+ Mix clones at E17.5 (Chi-square test: E17.5, *p* = 0.001; Figure 3b). This finding indicated that a significant reduction in proportions of Mix F/+ clones in response to *Egfr* deletion occurs late during gliogenesis (between E16.5 and E17.5). Moreover, it appears that early E11.5 derived F/+ clones are more likely to progress into gliogenesis following neurogenesis (leading to more Mix clones), which may lead to more G lineages observed at the later time points. Thus, it is unlikely that the progression into gliogenesis from neurogenesis occurs in a pre-determined subset of clones, but rather more clones can become gliogenic if a loss of glia is somehow sensed in clonally-related populations.

When analyzing total MADM neurons per clone (without separating green and red cells) in +/+ cortices, there was no significant difference between N and Mix clones at any time point (blue bars, Figure 3c). Neuron totals in F/+ N and Mix clones were also similar in E11.5 and E15.5 cortices, whereas N clones were absent at E16.5 and Mix clones were missing at E17.5 (Figure 3c). Interestingly, when we compared red and green neurons separately, the Mix F/+ clones at E11.5 produced significantly more red (*WT*) neurons than E11.5 +/+ Mix clones (Figure 3c; rank-sum test, *p* = 0.026), whereas N +/+ and N F/+ clones failed to produce significantly different numbers of red neurons at E11.5. In contrast, numbers of neurons per clone at E11.5 were similar between green F/+ (*Egfr-null)* and green +/+ (WT) cells in both N and Mix clones (Figure 3c; rank-sum test; *p* = 0.52 and *p* = 0.26, respectively). The neuronal numbers at later time points were small with fewer neuron-containing clones; however, significant differences between red and green populations of neurons were observed mostly in F/+ Mix clones (Figure 3c). Thus, *Egfr* deletion has no effect on neuronal expansion in green MADM clones, whereas the elevated expansion of red MADM glia coincides with significantly more red neurons in *F/+* lineages at E11.5. This finding suggests that elevated production of glia in the red MADM population coincides with a significant increase in neuronal output early during neurogenesis. 

Glial production was relatively consistent across clone types and MADM induction times in +/+ cortices (Figure 3d). G clones produced 12 ± 1 glia at E15.5, 13 ± 1 glia at E16.5, and 12 ± 1 at E17.5, and Mix clones produced 8 ± 1 glia at E11.5, 11 ± 2 glia at E15.5 and 12 ± 2 at E17.5, matching our past clonal analysis [33]. The exception to this consistency appeared in E16.5 +/+ Mix clones, which produced 27 ± 5 glia, a significantly larger number of glia than Mix +/+ clones at E15.5 and E17.5 (Wilcoxon Rank Sum Test, *p* < 0.05 for both), as well as E16.5 +/+ G clones (Wilcoxon Rank Sum Test, *p* < 0.01). Similar comparisons in F/+ cortices revealed that glial production was also consistent in G clones (18 ± 3 glia at E15.5, 21 ± 4 glia at E16.5, 24 ± 4 at E17.5; Figure 3d). Mix F/+ clones produced more glia at E16.5 than at E11.5 or E15.5, although the difference failed to reach significance, likely due to the large variance in the cell numbers (19 ± 3 glia at E11.5, 15 ± 5 glia at E15.5, 35 ± 10 at E16.5, Wilcoxon Rank Sum Test, *p* = 0.16 and *p* = 0.17, respectively). The difference between glial production in Mix F/+ and G F/+ clones at E16.5 was also not significant, unlike in the +/+ case (*p* = 0.20, Figure 3d). At E17.5, while some Mix clones were present in +/+ cortices, none were observed in F/+ cortices. 

Mix clones at E15.5 and E16.5 failed to produce a significantly different distribution of total glia per clone in response to deletion of *Egfr* in F/+ cortices, despite the decrease in glial production in the *Egfr-null* population (green) and its increase in *WT* clones (red) (Figure 3d; rank-sum test, E15.5 *p* = 0.41, E16.5 *p* = 0.66). However, Mix E11.5 F/+ clones contained an elevated number of glia as compared with Mix E11.5 +/+ clones (rank-sum test, *p* = 0.002), and E17.5 F/+ cortices contained no Mix clones. This suggests that the apparent compensatory overexpansion of red *WT* glia in response to *Egfr* deletion in their green siblings occurs early during the gliogenic switch. In contrast with the general behavior of Mix clones, G clones produced a larger average number of glia in F/+ than in +/+ clones at all time-points (E15.5, *p* = 0.047; E16.5, *p* = 0.026, E17.5, *p* = 0.0014; Figure 3d). While it is unclear why an increase in total glial production appears selective to G clones and not Mix clones, it is conceivable that Mix clones that produce most of their glia in the red sublineages give the robust appearance of “glial only” clones. Alternatively, the possibility that some G clones may originate from outside the cortical progenitor zones could explain their absence in E11.5 inductions and their differential response to *Egfr* deletion in later induction ages. 

### 3.3. Gliogenic Clones Expand Asymmetrically in the Dorsolateral Cortices

**Figure 4:**
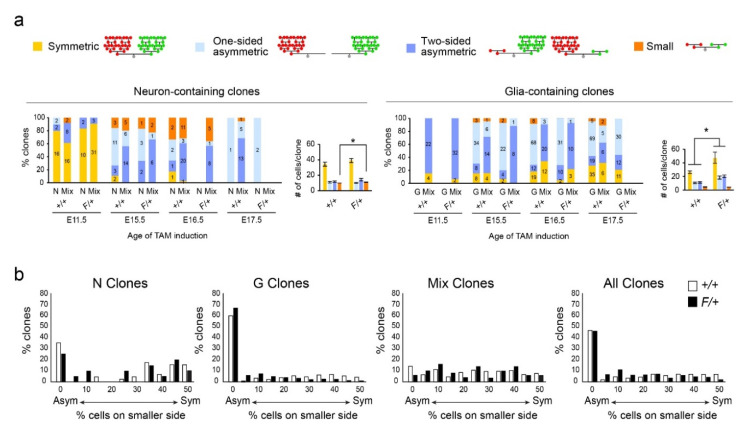
Analysis of clonal MADM data in Neuron-containing and Glia-containing clones in the dorsolateral cortices. (**a**) Analysis of symmetry in clones at different time points and genotypes. Data are percentages of clones that form the different categories of clones as indicated. Numbers in the bars indicate the numbers of clones that fall into each category. Charts present numbers of cells per clone for each category of neuron-containing and glia-containing clones for all time points combined. Data are mean ± sem. *, rank-sum test, *p* < 0.05. (**b**) Percentages of clones across a gradient of asymmetry depicted on the x-axes (0 = one-sided asymmetric; 50 = symmetric) in +/+ and F/+ cortices. Data are shown separately for each clone type and for all clones combined. The majority of G clones are one-sided asymmetric in both genotypes (green and red MADM cells are combined).

Given the power of MADM in delineating subclones derived from sibling progenitors with definitive genetic labels, we next assessed the asymmetry of clonal expansion during gliogenesis (Figure 4; Figure S4). First, we noted that a number of clones contained only red or only green cells (neurons and/or glia) with no cells found in the other lineage. We labeled these clones as “one-sided asymmetric.” Of the G and Mix clones with cells (either neurons or glia) in both red and green MADM lineages, we categorized each clone based on its numbers of red and green glia: “symmetric,” with >3 glia in both red and green lineages, “two-sided asymmetric,” either with >3 glia in one lineage and 1–3 in the other or with 0 glia in one lineage and >0 in the other, or “small” having 1–3 glia in both lineages (Figure 4a). The second definition of two-sided asymmetric clones does not make the clone one-sided asymmetric despite having zero glia in the other lineage; in this case, there were neurons in the lineage with zero glia. These categorizations were also applied to the red and green neurons in N and Mix clones; Mix clones thus were given two symmetry categories, one for their neurons and the other for their glia (separated in Figure 4a). Analyses of clone sizes, symmetries, and clone size distributions were then performed across different subsets of the data grouped or separated by MADM induction time, genotype, and clone type. 

For neuron-containing clones, we separated clones into categories by their symmetries and time points, then compared the neuron production of +/+ versus F/+ clones in each category (Figure 4a). The only significant difference between +/+ and F/+ neuron production was seen in small E16.5 clones (*p* = 0.0154). We note that neuron production in small clones, although minor, was significantly different between +/+ and F/+ when grouping all time points together (+/+, 3 ± 0.2 neurons; F/+, 4 ± 0.3 neurons, *p* < 0.01). Interestingly, when a similar comparison between +/+ and F/+ neuron production was performed for groups of clones separated by symmetry and clone type (N or Mix), the only significant difference was seen in small Mix clones (*p* < 0.01). These results suggest that neuron production within each symmetry group is comparable overall between +/+ and F/+ cortices, but *Egfr* deletion affects the output of small neuron-containing clones. Since small clones are likely the product of MADM-based labeling of a progenitor near the end of its lineage progression, these combined results suggest that *Egfr* deletion increases the capacity for neuron production at the end of neurogenesis. As a reminder, *Egfr* deletion results in the blockade of glial production while double dosage of *Egfr* in the same clone results in overproduction of glia (especially astrocytes) in the dorsolateral cortices. Thus, the effects on neurogenesis in small clones likely reflect early effects of *Egfr-null* progenitors on their WT siblings. 

We repeated the category-analysis for glia-containing clones in +/+ versus F/+ cortices. With all time points and clone types combined, the total glia per clone were significantly elevated in all categories of F/+ clones except in small clones relative to the same categories in +/+ clones (symmetric: 26 ± 2 vs. 47 ± 8, one-sided asymmetric: 10 ± 1 vs. 18 ± 2, two-sided asymmetric: 11 ± 1 vs. 20 ± 2, *p* < 0.01 in all cases; Figure 4a). We know from earlier analyses that this elevation is due to an increase in numbers of red *WT* MADM glia in F/+ cortices. When broken down by time, significant differences between glial production in +/+ and F/+ cortices were specifically observed in E16.5 symmetric, E15.5 and E17.5 one-sided asymmetric, and E11.5 and E17.5 two-sided asymmetric clones (*p* < 0.05 in all cases; Figure 4a). Small glia-containing clones produced similar total glia per clone in +/+ and F/+ cortices at all times. When examining +/+ versus F/+ clones by symmetry category and clone type (G and Mix), we found that while Mix clones were similar in their glial production in any of the symmetry groups, it was significantly different for symmetric, one-sided asymmetric, and two-sided asymmetric G clones (*p* < 0.05 in all cases). Thus, all symmetry categories of glia-containing clones, except the small category, were affected by the deletion of *Egfr* in the green MADM subclone and its double dosage in their sibling red *WT* MADM cells. The increase in glial production in each of these symmetry groups can specifically be tied to G clones rather than Mix clones. To examine this further, rather than categorizing clones into symmetry categories based on absolute numbers of cells, we plotted clonal symmetries using percentages of cells in each of the clone’s smaller subclone (0–50%). The results showed a tendency for more asymmetric distributions of cells between subclones, particularly in G clones (Figure 4b).

Taken together, the findings from our clonal symmetry analyses indicate that unlike neurogenesis, gliogenesis is highly asymmetric at the clonal level, resulting in highly variable distributions of clone types and sizes. In contrast to its effects on neurogenesis, *Egfr* deletion in our MADM labeling paradigm and dosage model increases average overall glial output in the red F/+ MADM subclones, but has no discernible effect on clones that have only one or two remaining divisions.

### 3.4. Gliogenic Clones Exhibit a Stochastic Mode of Expansion in the Dorsolateral Cortices

**Figure 5:**
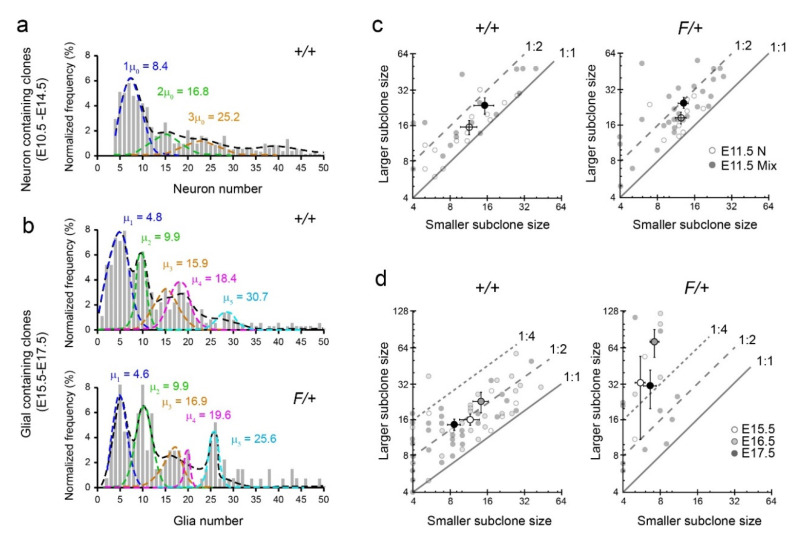
Gliogenesis exhibits a stochastic pattern of clonal expansion in the cortex. (**a**) Gaussian curve fitting for analysis of size distribution of clones versus their normalized frequency for early neurogenic clones (E10.5–E14.5). Values for the histogram and Gaussian curves were extracted manually using the WebPlotDigitizer (https://automeris.io/WebPlotDigitizer/citation.html) from Gao et al. [1]. (**b**) Gaussian curve fitting for analysis of size distribution of clones versus their normalized frequency for gliogenic clones (E15.5–E17.5). (**c**) Scatterplot presenting the size (number of cells) of larger versus smaller sibling subclones of individual clones during the neurogenic period at E11.5 in +/+ and F/+ cortices. (**d**) Scatterplots presenting the size (number of cells) of larger versus smaller sibling subclones of individual clones during the gliogenic period in +/+ and F/+ cortices. White, black, and gray dots with error bars represent mean ± sem, faded dots indicate individual clones, and the slopes indicate ratio boundaries as indicated in both (**c**,**d**).

Given the differences between neurogenesis and gliogenesis and the robust changes in gliogenesis from E15.5 to E17.5, we next established criteria to categorize clonal expansion in our MADM data sets as deterministic or stochastic. Rather than mathematically defining this difference by setting arbitrary thresholds for degrees of variability in number of cells produced, we adopted the assumption that the two processes are mutually exclusive. Therefore, we can define stochastic events as “not deterministic” and datasets that fail to reveal the hallmark mathematical patterns of a deterministic mechanism can be assigned as stochastic. Previous studies have noted that patterns in the distribution of clone sizes (i.e., histogram of total neurons or total glia per clone) can indicate deterministic or stochastic patterns at the clonal level. For example, the distribution of neurons produced in a stochastic model with assigned probabilities for types of cell division (Galton–Watson process) has a shape similar to a geometric distribution, with the specific scaling of the distribution determined by the cell division probabilities [38]. On the other hand, the proposed deterministic process of neurogenesis was associated with a multi-Gaussian distribution whose peaks are equally spaced (Figure 5a; [1]). We therefore considered several models as possible matches for the distribution of glia per clone in our E15.5, E16.5 and E17.5 induction data: a one-stage stochastic model [38], a more complex two-stage stochastic model, a multi-Gaussian with equally spaced peaks, and a multi-Gaussian with unequally spaced peaks. For each of these models, we tested their goodness of fit to several glial clone size distributions: +/+ G clones, +/+ Mix clones, +/+ G and Mix clones, and the same for the F/+ genotype, considering only clones containing 50 or less glia.

We found that a one-stage stochastic model sufficiently represented the distribution of +/+ G clones, whereas the deterministic equally-spaced multi-Gaussian sufficiently represented +/+ Mix clones. Interestingly, the distribution of +/+ G and Mix clones was not represented well by the stochastic models or the equally-spaced multi-Gaussian, but instead the best fit was produced by the multi-Gaussian with unequally spaced peaks (Figure 5b). The uneven spacing of frequent glial clone sizes indicates a lack of predictable behavior across the full population of gliogenic clones—we label this pattern as stochastic. Applying the same multi-Gaussian curve fitting to the F/+ data revealed that there was not a large difference in the small glial clone sizes compared to the +/+ data, as measured by the location of the first two Gaussian peaks, but the larger clones contribute to the difference between the clonal output of the two genotypes (Figure 5b).

We also took a second approach in identifying deterministic or stochastic patterns by examining the clonal symmetries for fractal characteristics (Figure 5c,d). In neurogenesis, it was shown that the ratio of neurons in the larger to smaller subclone was preserved over time for “symmetric” clones having four or more neurons in each subclone [1]. By extension, since clone sizes decreased predictably over successive MADM induction points during neurogenesis, this ratio was also preserved over total clone size. We note that this self-similarity of clonal symmetries throughout neurogenesis is characteristic of fractal patterns. Interestingly, the preserved ratio during neurogenesis was ~1.6 (Figure 5c), corresponding to the golden ratio and hinting at clonal proliferation capacity being described by Fibonacci numbers [46]. This pattern was observed for neuron production in both E11.5 +/+ and F/+ clones (Figure 5c). In contrast, we found that gliogenic clones categorized as symmetric (>3 glia in both red and green sublineages) tended to have a wider range of relative symmetry ratios between the larger and smaller sublineages, often greater than 2:1. Additionally, we found that larger symmetric gliogenic clones (>25 total glia with at least 4 glia on each side) tended to have greater ratios of larger to smaller subclones than smaller symmetric gliogenic clones (<12 total glia with at least 4 glia on each side) in both +/+ and F/+ cortices (Figure 5d). Thus, unlike neurogenesis, subclone ratios were not preserved between larger and smaller gliogenic clones, with the smaller symmetric gliogenic clones tending to approach more consistent ratios between 2:1 and 1:1. This suggests far more stochasticity in MADM subclone fate during the expansion phase of gliogenesis, with a possible shift to more predictable symmetric divisions as a clone reaches the end of gliogenesis. Thus, the lack of preserved symmetries in gliogenic clones does not point to the level of organization seen in the fractal patterns of neurogenesis. This level of disorganization was even more profound in F/+ cortices, where unusually large red glia-containing MADM clones are present (Figure 5d). Taken together, our multiple analytical approaches suggest that while clonal neurogenesis resembles a deterministic pattern of expansion, gliogenesis may lack the cell intrinsic machinery that dictates defined numbers of divisions at the level of individual clones.

## 4. Discussion

**Figure 6:**
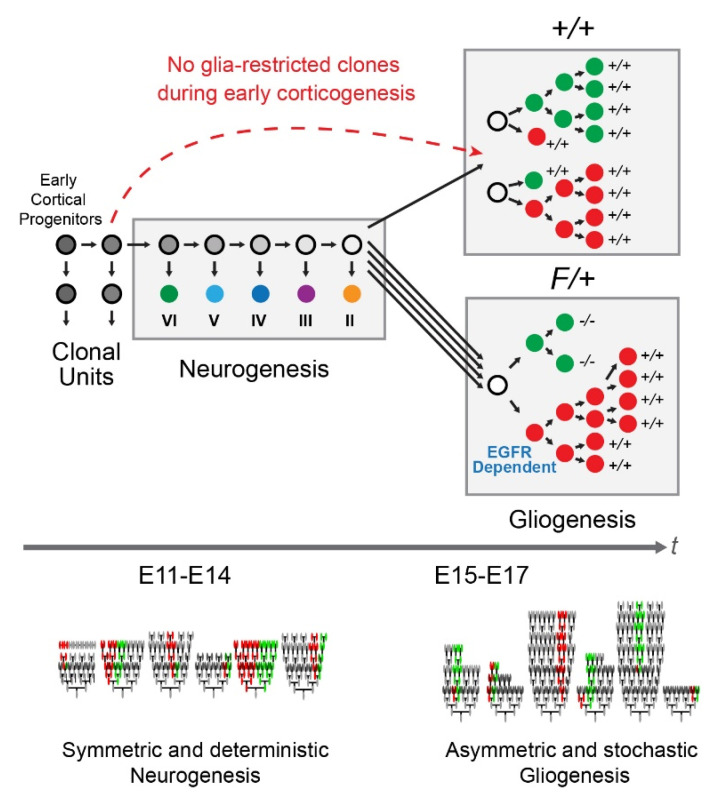
Summary: MADM enabled labeling of sparse clones in dorsolateral cortices of +/+ and F/+ mice reveals switch to gliogenesis around E15 followed by seeding of glia without involvement of glia-restricted clones from early neurogenic period. Allelic Egfr dosage analysis using MADM with a conditional allele for Egfr revealed that the background cells and their genotype (*Egfr-heterozygous*) influence *Egfr-null* (green) and wild type (*WT*, red) MADM siblings. Comparison of cortical MADM progeny in +/+ and F/+ cortices exposed a dosage response in glial populations whereby *WT* MADM cells expand to compensate for loss of glia in their MADM green siblings. Neurogenic clones exhibit symmetric and deterministic patterns of expansion, whereas clonal expansion during gliogenesis is highly asymmetric and stochastic.

Figure 6 is a summary of our current findings. Our results indicate that clonally derived glial cells in the cortical parenchyma (layers II–VI) largely consist of astrocytes with only few oligodendrocytes in the deep layers of the P30 mouse dorsolateral cortex. This finding does not rule out the possibility that oligodendrocytes and their precursors occupy the upper layers of the cortex earlier during postnatal maturation. Such distribution patterns would require that oligodendrocytes either die, migrate away from the maturing cortical layers, and/or transdifferentiate into astrocytes or other cell types following their function as oligodendrocytes. In fact, several waves of proliferation during and after the switch from neurogenesis to gliogenesis give rise to distinct populations of oligodendrocytes, with the early population (which is likely derived from the ventral telencephalon) disappearing during early postnatal periods [47,48]. Moreover, as indicated in the results, we excluded SEZ/WM cells from our analysis since the cellular populations in that tissue are highly complex and require detailed analyses with the aid of molecular markers and additional induction time points during early postnatal days. In addition, given that cells may continue to migrate tangentially in the SEZ/WM into early adulthood, clonality of cells in this tissue must be established using additional approaches such as genetic bar-coding methods (e.g., [49]). As an example, the ependymal cells found in our clones were extremely small in number and often appeared as pairs, suggesting their derivation from terminal symmetric divisions. However, given the likely planar expansion of the ependymal layer relative to the overlying WM and cortical plate, it is possible that many clonally-related ependymal cells were missed in our analysis and should be reexamined in ependymal wholemounts, possibly in combination with the use of bar-coding methods.

We also found high diversity in the total cellular output and division patterns of glia-containing clones in dorsolateral cortices. Clones containing only glia appear during the gliogenic period and are not present in the early developing cortex, suggesting the absence of glia-restricted clones during early corticogenesis. The employment of MADM was instrumental as we found that the observed types of glial clones are effected by the deletion of *Egfr* in one clonal sibling, resulting in cell-autonomous loss of gliogenic potential. Interestingly, the clonal siblings to the *Egfr-null* progenitors that have double the dosage of *Egfr* relative to background non-MADM cells appear to somehow sense the loss of *Egfr* either through cell–cell interactions or through cell competition mechanisms. This compensation results not only in the balancing out of total populations of glia in the cortex, but also appears to over-expand beyond what is seen in +/+ cortices. The precise mechanisms underlying the relationship between sibling pairs of progenitors remain to be determined, although a past study has suggested the existence of physical interactions (e.g., gap junctions) between pairs of sibling progenitors in the developing cortex [50].

Our findings also reveal that glial production was consistent over time during embryonic development, except at E16.5. This suggests the absence of a defined period of significant symmetric proliferation during gliogenesis, similar to the early period of symmetric expansion during cortical neurogenesis. It was surprising that the MADM cells induced at E11.5 or E15.5 failed to capture the significant elevation in glial production documented in E16.5 Mix clones. That is, since MADM should theoretically track all cells in a lineage after the initial MADM-recombined cell divides, evidence for later clonal behaviors in earlier labeled lineages was expected if all later clones are descendants from earlier ones. As such, it remains unclear whether later gliogenic lineages are descended from ones labeled earlier with MADM, which may suggest that some later clones (E16.5 and E17.5) may have originated from outside the cortex (e.g., the ventral telencephalon) and thus were not observed in earlier inductions.

Finally, we provided evidence that gliogenic expansion occurs in a stochastic manner in individual clones, which is in contrast to the predictable and deterministic neurogenic clonal expansion that occurs earlier during cortical development [1]. Specifically, the distribution of glia per clone was consistent with a multi-Gaussian model with unequally spaced peaks. The latter property of our model supports the notion of stochastic gliogenic expansion, in contrast to the simpler set of underlying clonal expansion rules and equally spaced population-level multi-Gaussian peaks exhibited in neurogenic clonal expansion [1]. The biological significance of this stochasticity remains unclear at this juncture; however, it is tempting to compare it to the variability reported for clonal expansion in non-neuronal tissues that can regenerate throughout life [2,3,4,5]. The mechanisms that underlie the stochasticity inherent to glial expansion and how it differs from deterministic patterns of neurogenic clonal expansion remain to be determined. Whether clonal principles described in the current study apply to pathogenic gliogenesis in the damaged or diseased cortex at various ages remains to be thoroughly studied.

## Supplementary Materials

The following are available online at https://www.mdpi.com/2073-4409/9/12/2662/s1, Figure S1: Astrocyte versus oligodendrocyte fates in MADM clones; Figure S2: Proportional distribution of lineage-restricted and multi-lineage clones in the +/+ and F/+ cortices; Figure S3: Laminar distribution of glial types; Figure S4: Analysis of asymmetry in MADM clones during gliogenesis segregated by green and red MADM cells.
