# Supplementary material for: Clonal Analysis of Gliogenesis in the Cerebral Cortex Reveals Stochastic Expansion of Glia and Cell Autonomous Responses to Egfr Dosage
Source: Cells. 2020 Dec 11;9(12):2662. doi: 10.3390/cells9122662 (PMC7764668; doi:10.3390/cells9122662)
Supplement: Supplementary file 1 [file cells-09-02662-s001.pdf]

cells

MDPI

**Supplementary Figures**

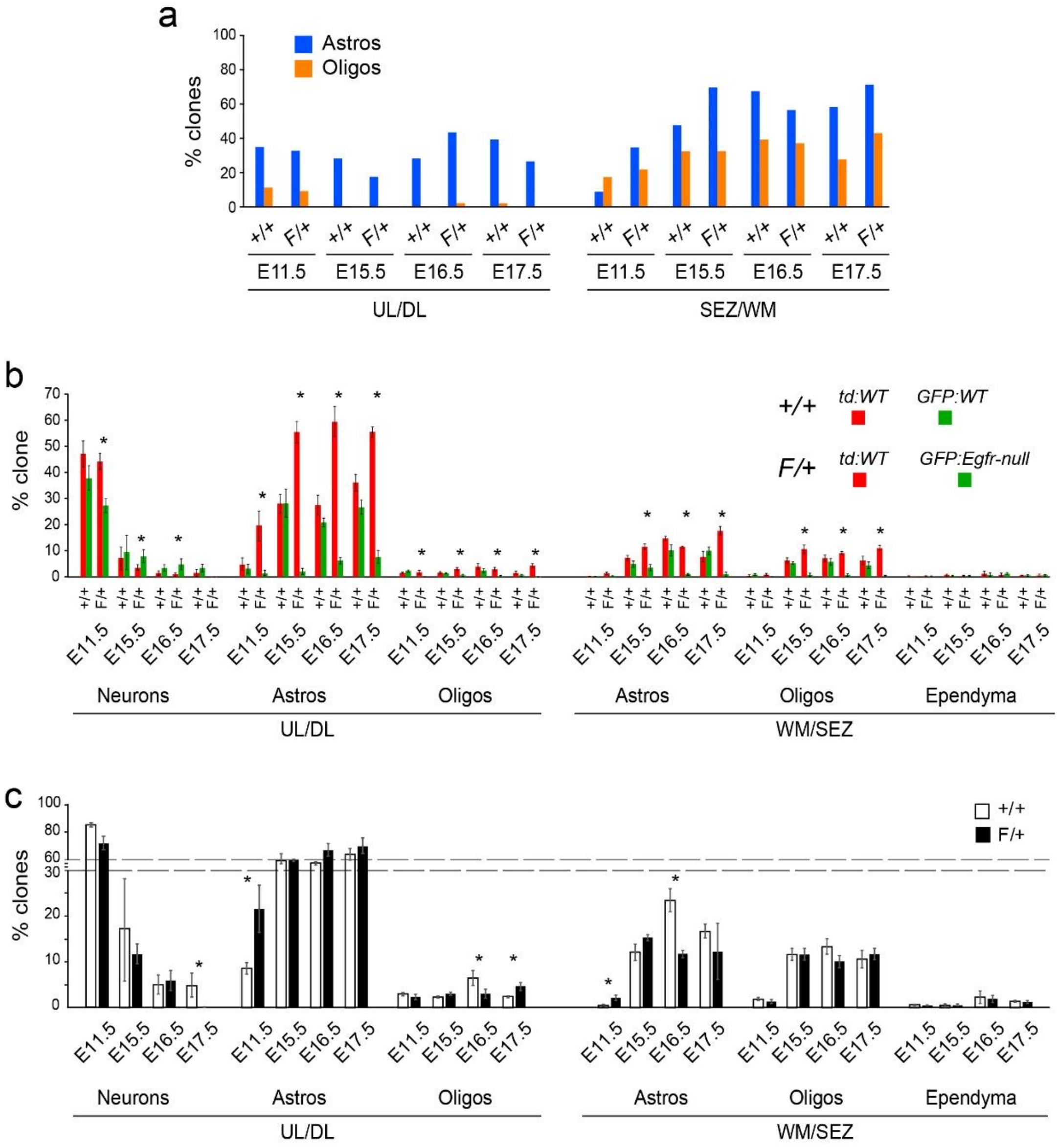


**Figure S1. Astrocyte versus oligodendrocyte fates in MADM clones.** (**a**) Percentages of MADM clones containing astrocytes and/or oligodendrocytes in the UL/DL versus SEZ/WM of dorsolateral cortices at various induction times and genotypes. **(b)** Percentages of cell types in +/+ and F/+ cortices at different induction times broken down by red and green MADM distributions in the UL/DL or SEZ/WM of the cortex as indicated. **(c)** Percentages of cell types in +/+ and F/+ cortices at different induction times with red and green MADM distributions combined in the UL/DL or SEZ/WM of the cortex as indicated. Data are mean ± sem. *, Student’s t-test, $P<0.05$.

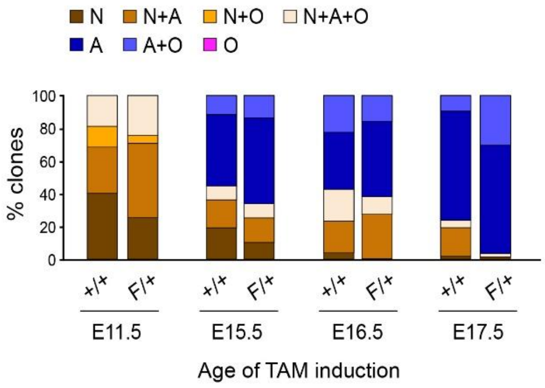


**Figure S2. Proportional distribution of lineage-restricted and multi-lineage clones in the +/+ and F/+ cortices.** Percentages of clones containing only neurons (N), astrocytes (A), or oligodendrocytes (O) in layers I-VI of the cortex. Data does not include cells in the SEZ/WM. Note no O clones were found at any age or genotype. Multi-lineage clones are presented as indicated.

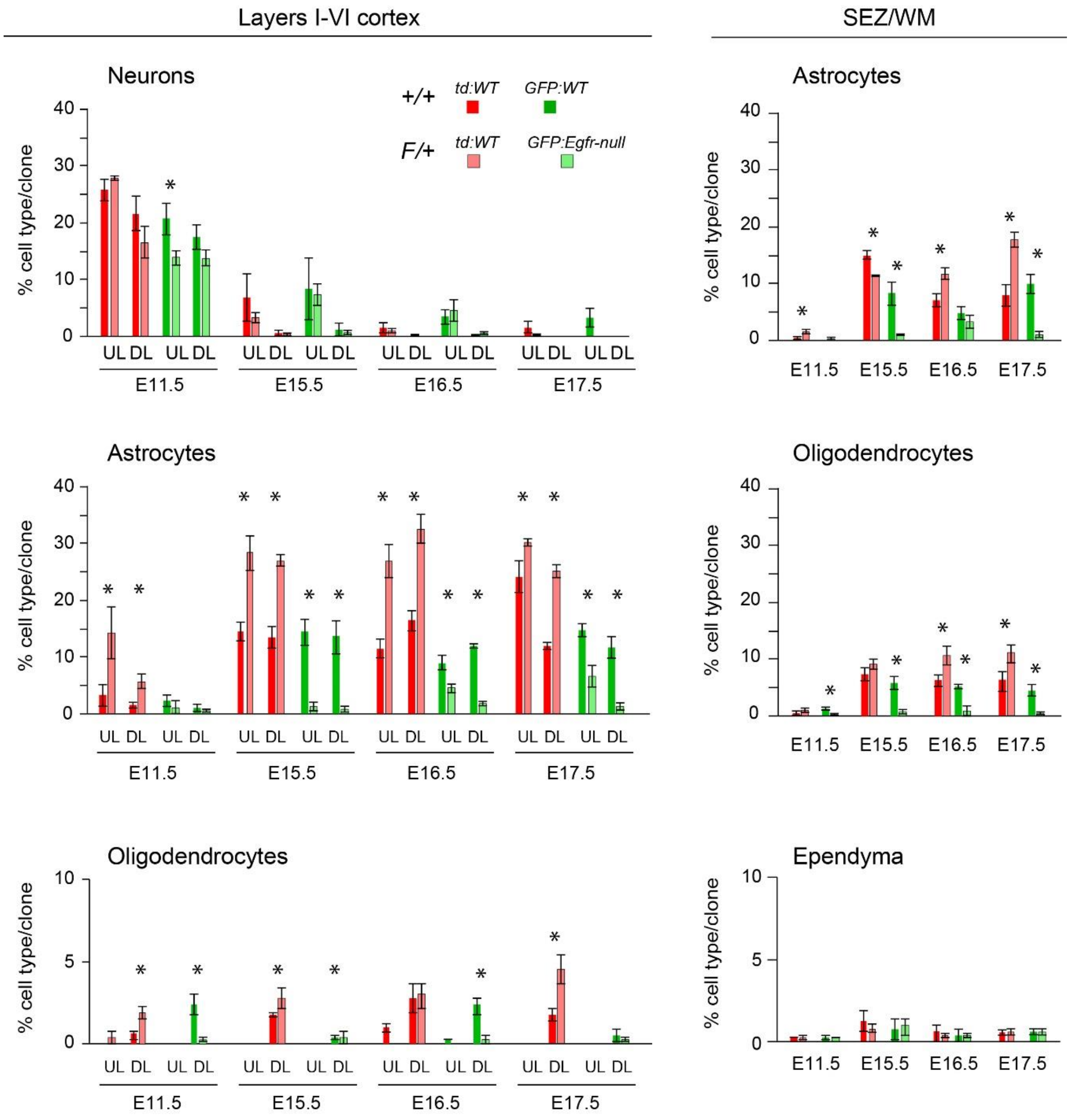


**Figure S3. Laminar distribution of glial types.** Charts illustrating average percentages of cell types per clone in the upper layers (UL), deep layers (DL) and the SEZ/WM of the cortex broken down by red and green MADM cells in +/+ and F/+ dorsolateral cortices. Data are mean ± sem. *, Students t-test, $P<0.05$.

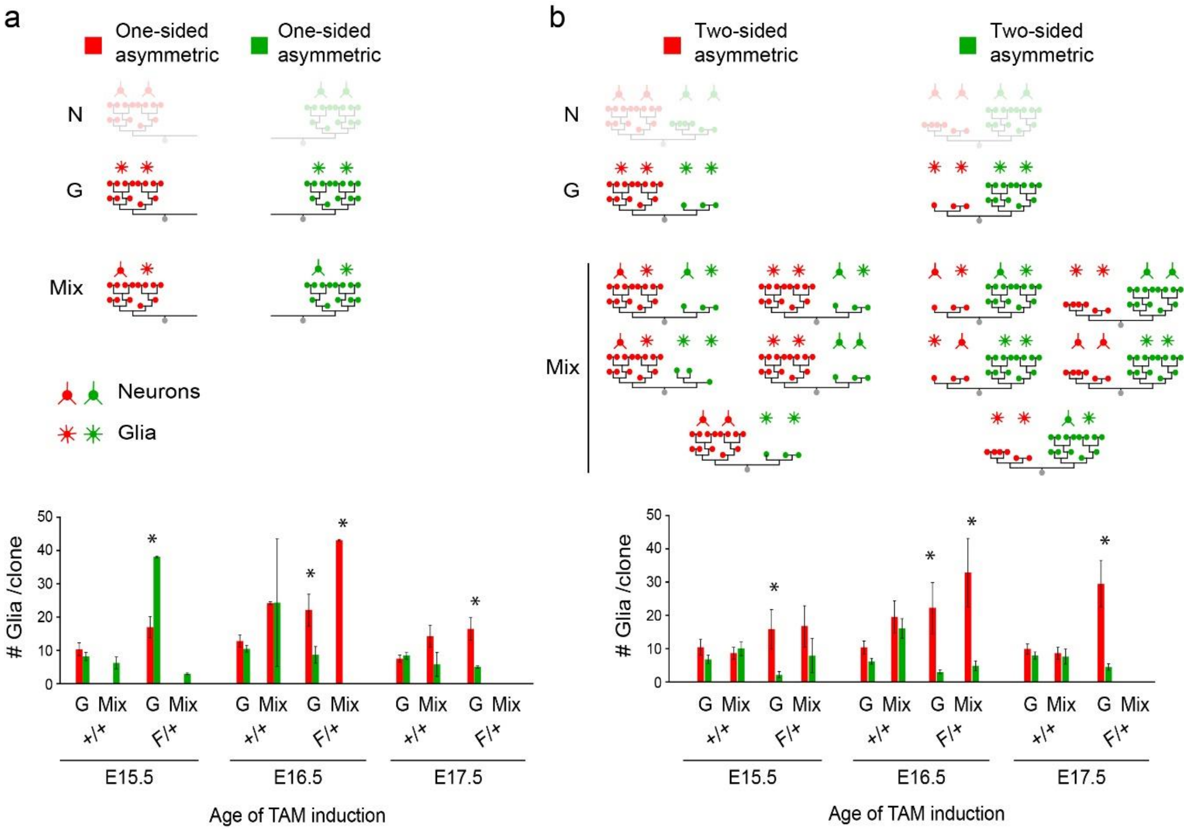


**Figure S4. Analysis of asymmetry in MADM clones during gliogenesis segregated by green and red MADM cells.** N clones were excluded since none were observed during the gliogenic period. Representative patterns of different clone types are depicted above for **(a)** one-sided asymmetric, and **(b)** two-sided asymmetric clones. Charts present average numbers of glia per clone in different clone types and genotypes. Data are mean ± sem. *, rank Sum test, $P<0.05$.
